# Vitamin D Supplementation on Detailed Fall Characteristics

**DOI:** 10.1093/geroni/igaa057.2738

**Published:** 2020-12-16

**Authors:** Amal Wanigatunga, Alice Sternberg, Amanda Blackford, Yurun Cai, Jennifer Schrack, Edgar Miller, David Roth, Lawrence Appel

**Affiliations:** 1 Johns Hopkins Bloomberg School of Public Health, Baltimore, Maryland, United States; 2 Johns Hopkins School of Public Health, Baltimore, Maryland, United States; 3 Johns Hopkins School Of Medicine, Baltimore, Maryland, United States; 5 Johns Hopkins University, Baltimore, Maryland, United States; 6 Johns Hopkins University School of Medicine, Baltimore, Maryland, United States

## Abstract

Evidence suggests Vitamin D supplementation may reduce fall risk in older adults, but effects on fall location and severity are less well described. We used STURDY trial data to examine whether Vitamin D supplementation reduces indoor, outdoor, “consequential” (falls resulting in injury or medical care), and repeat fall risk. Participants (77[SD=5.4] years; 44% women) were randomized to receive 200 (n=339) or 1000IU/day (n=349) of vitamin D3. Indoor, outdoor and consequential fall rates were similar between the ≥1000IU/day and 200IU/day groups (rate ratio [RR]:1.22, 95%CI:0.96-1.55; RR:0.85, 95%CI:0.65-1.10; and RR:1.16, 95%CI:0.93-1.45, respectively) during follow-up. The proportion of repeat fallers was similar between ≥1000IU/day versus 200IU/day groups over 3 months (7.8%[27/346] versus 6.5%[22/336], p=0.22), 6 months (18.8%(60/319) versus 16.2%(51/315), p=0.40), 12 months (29.9%(81/271) versus 31.2%(84/269), p=0.78) and 24 months (48.2%(66/137) versus 49.6%(66/133), p=0.90). In conclusion, Vitamin D supplementation ≥1000IU/day did not reduce indoor, outdoor, consequential or repeat fall risk.

